# Genetic variants in miR-146a and miR-196a2 in endometriosis: a Brazilian study

**DOI:** 10.1590/1806-9282.20231382

**Published:** 2024-05-17

**Authors:** Gabriela Caramano de Oliveira, Mariangela Torreglosa Ruiz Cintra, Marco Fábio Prata Lima, Mariana Kefalas Oliveira Gomes, Alessandra Bernadete Trovó de Marqui

**Affiliations:** 1 Universidade Federal do Triângulo Mineiro – Uberaba (MG), Brazil.; 2 Universidade Federal do Triângulo Mineiro, Institute of Exact, Natural Sciences and Education – Uberaba (MG), Brazil.; 3 Universidade Federal do Triângulo Mineiro, Institute of Health Sciences – Uberaba (MG), Brazil.; 4 Universidade Federal do Triângulo Mineiro, Institute of Biological and Natural Sciences – Uberaba (MG), Brazil.

**Keywords:** Endometriosis, MicroRNAs, Real-time polymerase chain reaction, Biomarkers, Polymorphism, genetic, Diagnosis

## Abstract

**OBJECTIVE:**

The aim of this study was to determine the allelic and genotypic frequencies of the polymorphisms, rs2910164 miR-146a and rs11614913 miR-196a2, by investigating their association with endometriosis.

**METHODS:**

This is a case–control study performed with approximately 120 women. The polymorphisms were determined by real-time polymerase chain reaction. For the statistical analysis, the chi-square and logistic regression tests were used.

**RESULTS:**

There were no significant differences in the genotype and allele frequencies of rs2910164 and rs11614913 between cases and controls. The frequencies in both polymorphisms are in accordance with Hardy-Weinberg equilibrium regarding miR-146a (patients: χ^2^=1.64, p=0.20; controls: χ^2^=0.25, p=0.62) and miR-196a2 (patients: χ^2^=0.58, p=0.44; controls: χ^2^=2.78, p=0.10). No relationship was observed between rs2910164 and rs11614913 and endometriosis in the inheritance models analyzed.

**CONCLUSION:**

In this study, our results show that the studied polymorphisms are not implicated in the development of endometriosis.

## INTRODUCTION

Endometriosis is a gynecological condition defined by the presence of endometrial tissue in extrauterine locations. The clinical presentation is extremely variable with symptoms including dysmenorrhea, dyspareunia, pelvic pain, dyschezia, dysuria, changes in bowel habits, and often infertility^1^. The disease has a negative effect on quality of life and may cause psychological disorders and lower productivity at work^2,3^.

Currently, videolaparoscopy and subsequent anatomopathological analysis are still present as the gold standard procedure for the definitive diagnosis of endometriosis, resulting in delayed diagnosis of 8–12 years. Therefore, the search for noninvasive diagnostic test options has been the subject of intensive investigation in the scientific literature^4,5^. In this sense, genetic polymorphism deserves to be highlighted, especially single nucleotide polymorphisms (SNPs), such as in regions that encode microRNAs, a class of noncoding small RNAs responsible for the post-transcriptional regulation of gene expression and that may be implicated in the pathophysiology of endometriosis^6,7^. A recent study suggests that four miRNAs could be included as a prognostic marker in endometriosis^7^. However, future clinical studies should evaluate the efficacy of these miRNAs in endometriosis diagnosis and treatment^8^.

It is also worth noting that miRNAs can control inflammatory responses, cell proliferation, angiogenesis, and tissue remodeling, which are common biological processes in endometriosis^9^. A recent study evaluated the prevalence of miRNA variants, such as miR-146a rs2910164, miR-149 rs2292832, miR-196a2 rs11614913, and miR-499 rs3746444, in endometriosis^10^. Some studies have investigated the association of rs2910164 and rs11614913 in female reproductive disorders such as polycystic ovary syndrome^11-14^, preeclampsia^15,16^, ovarian cancer^17^, and idiopathic recurrent pregnancy loss^18^. However, less is known about the effect of these miRNAs polymorphisms in endometriosis^10,19^, which highlights the need for further research.

Hence, the objective of the present study was to determine the allelic and genotypic frequencies of the polymorphisms rs2910164 miR-146a and rs11614913 miR-196a2 and investigate their association with endometriosis.

## METHODS

This is a case–control study approved by the Research Ethics Committee of the Federal University of Triângulo Mineiro (UFTM), protocol 1628. All participants who signed the informed consent form were from the Gynecology and Obstetrics outpatient clinic at UFTM, Brazil. The presence (case group) or absence (control group) of the disease was confirmed through videolaparoscopy or laparotomy. The women with endometriosis (case group) had histological confirmation of endometriosis and classification of the disease according to the Revised American Society for Reproductive Medicine. The control group included endometriosis-free patients who underwent surgery for tubal sterilization, chronic pelvic pain, and infertility. Participants’ inclusion in the study occurred in the period from 2012 to 2016.

It is worth noting that the gold standard for diagnosing endometriosis is done through a surgical procedure called laparoscopy. As it is an invasive method, it is only justified for women with clinical complaints associated with endometriosis. Therefore, to minimize the confounding clinical effects of the disease, samples from the control group were collected only after the histopathological report was issued following laparoscopy surgery, ruling out endometriosis.

Genomic DNA was extracted by a salting-out method^20^ from the peripheral blood drawn by venipuncture from the participants. Genotyping of the rs2910164 polymorphism was performed in 122 women (47 with endometriosis and 75 without the disease). A total of 115 (46 case group and 69 control group) were analyzed for the polymorphism miR-196a2 rs11614913.

The polymorphisms were determined by real-time PCR with the method of allelic discrimination on the SNP Genotyping Assay (Applied Biosystems). Primers and probes for the detection of rs2910164 miR-146a and rs11614913 miR196a2 polymorphisms are available from the manufacturer (Assay ID—C_15946974_10 and C_31185852_10, respectively). The reactions were carried out in 96-well plates, in the equipment StepOne™ Real-Time PCR System. Genotypes were determined from the results of amplification products observed as amplification curves recognized from the marking for each probe (VIC/FAM).

The statistical analysis used the chi-square test (χ^2^) to compare allele and genotype frequencies between the groups and to assay Hardy-Weinberg equilibrium. These analyses were carried out by the BioEstat program. Analyses were also performed assuming recessive, codominant, and dominant models of inheritance by the SNPStats program and odds ratio (OR), their 95%CI ranges by logistic regression adjusted for age, with p<0.05 being considered statistically significant.

## RESULTS

Allele frequencies and genotype of miR-146a rs2910164 and miR-196a2 rs11614913 polymorphisms are shown in Table 1. There were no significant differences in the genotype and allele frequencies of rs2910164 and rs11614913 between cases and controls.

**Table 1:** Frequency distribution of miR-146a and miR-196a2 genotypes and alleles in endometriosis patients and controls.

rs2910164 polymorphism	Patients (n=47)	Controls (n=75)	p
Genotypes	n (%)	n (%)	0.68
GG	26 (55.3)	41 (54.7)	
GC	20 (42.5)	30 (40)	
CC	1 (2.2)	4 (5.3)	
Alleles			0.87
G	0.77	0.75	
C	0.23	0.25	
**rs11614913 polymorphism**	**(n=46)**	**(n=69)**	**p**
Genotypes	n (%)	n (%)	0.26
CC	14 (30.5)	26 (37.7)	
CT	25 (54.3)	27 (39.1)	
TT	7 (15.2)	16 (23.2)	
Alleles			1.00
C	0.58	0.57	
T	0.42	0.43	

The frequencies in both polymorphisms are in accordance with Hardy-Weinberg equilibrium regarding miR-146a (patients: χ^2^=1.64, p=0.20; controls: χ^2^=0.25, p=0.62) and miR-196a2 (patients: χ^2^=0.58, p=0.44; controls: χ^2^=2.78, p=0.10).

The genotype of patients and controls were adjusted for age according to the inheritance models and no relationship was observed between rs2910164 and rs11614913 and endometriosis in the models analyzed (Table 2).

**Table 2:** Association of co-dominant, dominant, and recessive of miR-146a and miR-196a2 genotypes in endometriosis patients and controls.

rs2910164 polymorphism	Patients (n=43)	Controls (n=62)	OR (95%CI)	p
Codominant	n (%)	n (%)		0.94
GG	24 (55.8)	33 (53.2)	1.00	
GC	18 (41.9)	27 (43.5)	1.09 (0.49–2.42)	
CC	1 (2.3)	2 (3.3)	1.45 (0.12–16.98)	
Dominant				0.79
GG	24 (55.8)	33 (53.2)	1.00	
GC-CC	19 (44.2)	29 (46.8)	1.11 (0.51–2.43)	
Recessive				0.78
GG-GC	42 (97.7)	60 (96.8)	1.00	
CC	1 (2.3)	2 (3.2)	1.40 (0.12–15.94)	
**rs11614913 polymorphism**	**(n=43)**	**(n=62)**	**OR (95%CI)**	**p**
Codominant	n (%)	n (%)		0.32
CC	13 (30.2)	24 (38.7)	1.00	
CT	23 (53.5)	24 (38.7)	0.57 (0.23–1.08)	
TT	7 (16.3)	14 (22.6)	1.08 (0.35–3.36)	
Dominant				0.37
CC	13 (30.2)	24 (38.7)	1.00	
CT-TT	30 (69.8)	38 (61.3)	0.69 (0.30–1.57)	
Recessive				0.42
CC-CT	36 (83.7)	48 (77.4)	1.00	
TT	7 (16.3)	14 (22.6)	1.50 (0.55–4.10)	

The discrepancies in relation to the number of cases and controls presented in Table 1 are due to non-real-time amplification of some samples or the samples are over. In Table 2, there is a lack of information about age.

## DISCUSSION

The polymorphisms analyzed in miRNAs were not associated with endometriosis in the studied population. However, the literature explores the possible therapeutic strategies of miRNAs for the diagnosis and treatment of several human diseases, such as cancer^21^, diabetes mellitus^22^, and neurodegenerative conditions^23^.

Several regulatory mechanisms control the expression, activity, and bioavailability of miRNAs, among which the genetic polymorphisms can alter the expression pattern in genes involved in the development of specific pathologies^24^. A recent review showed that miR-126, miR-143, and miR-146b polymorphisms have been associated with risk of endometriosis; thus, understanding the role of these transcripts is a possible way to develop novel diagnostic tests and therapeutic targets for this disorder^9^.

In the present study, the polymorphisms rs2910164 and rs11614913 located on chromosomes 5 and 12, respectively, were analyzed. The base exchange observed in the miR-146a polymorphism was the substitution of C to G, decreasing the production of miR-146a^15^, while in the miR-196a2 polymorphism there was an exchange of C for T^13^. These polymorphisms were investigated in several gynecological conditions, with quite different results (Table 3).

**Table 3:** Summary of the mains results of previous studies that investigated rs2910164 and rs11614913 polymorphisms in female reproductive disorders.

Study	Country	Subjects	Gynecological condition analyzed	Polymorphism(s)	Association with the gynecological condition analyzed
Chang et al.^19^	Taiwan	218 cases–202 controls	Endometriosis	miR-196a2 rs11614913	miR-196a2=yes
Farsimadan et al.^10^	Iran	260 cases–260 controls	Endometriosis	miR-146a rs2910164	miR-146a=yes
				miR-196a2 rs11614913	miR-196a2=no
Hosseini et al.^11^	Iran	205 cases–205 controls	Polycystic ovary syndrome	miR-146a rs2910164	miR-146a=yes
Ebrahimi et al.^12^	Iran	180 cases–192 controls	Polycystic ovary syndrome	miR-146a rs2910164	miR-146a=yes
Li et al.^13^	Iran	385 cases–385 controls	Polycystic ovary syndrome	miR-146a rs2910164	miR-146a=yes
				miR-196a2 rs11614913	miR-196a2=yes
Soyman et al.^14^	Turkey	50 cases–50 controls	Polycystic ovary syndrome	miR-146a rs2910164	miR-146a=yes
Salimi et al.^15^	Iran		Preeclampsia	miR-146a rs2910164	miR-146a:
		Blood: 219 cases–242 controls			Blood=yes
		Placental: 111 cases–119 controls			Placental=yes
Asadi-Tarani et al.^16^	Iran		Preeclampsia	miR-196a2 rs11614913	miR-196a2:
		Blood: 315 cases–317 controls			Blood=no
		Placental: 103 cases–133 controls			Placental=yes
Lukács et al.^17^	Hungary	75 cases–75 controls	Ovarian cancer	miR-146a rs2910164	miR-146a=no
				miR-196a2 rs11614913	miR-196a2=no
Alipour et al.^18^	Iran	120 cases–90 controls	Idiopathic recurrent	miR-146a rs2910164	miR-146a=yes
			pregnancy loss	miR-196a2 rs11614913	miR-196a2=yes
Present study	Brazil	47/46 cases	Endometriosis	miR-146a rs2910164	miR-146a=no
		75/69 controls		miR-196a2 rs11614913	miR-196a2=no

According to Table 3, eight previous studies investigated miR-146a and six analyzed miR-196a2 polymorphism, among which one and three, respectively, found no association with endometriosis, in agreement with our results. Table 3 presents 11 studies on the miR146a polymorphisms, mainly, and miR196a2, seven of which were conducted in Iran. In all these studies, such polymorphisms were associated with the investigated conditions.

Positive results were found mainly in studies conducted in Iran (Table 3). The ethnicity effect might be related to differences in susceptibility to these polymorphisms. However, the present study did not collect information on ethnicity or skin color, and all individuals were from the same region of Brazil. The analysis of the mode of inheritance (Table 2) did not reveal differences between the groups in its distribution.

The studies in Table 3 used blood as a standard biological sample and techniques of polymerase chain reaction–restriction fragment length polymorphism (PCR-RFLP) and quantitative real-time polymerase chain reaction (RT-qPCR) to investigate these two polymorphisms. In this sense, we believe that such variables would not interfere with the results obtained.

Only two studies investigated these polymorphisms in endometriosis^10,19^, with different results for the miR-196a2. Genome-wide association studies have revealed 23 genome-wide significant loci that are associated with the risk of endometriosis, particularly on chromosome 12, where the miR-196a2 polymorphism is located^4^. Our results and number of individuals investigated were similar to those of Lukács et al^17^.

A systematic review concludes that no particular miRNA or miRNA combination has been validated for improved diagnosis of endometriosis to date. This may have reflected the heterogeneity of the disease and resultant differences in tissue composition^25^.

It was not possible to analyze the clinical data with the molecular ones, but it is noteworthy that, in this retrospective study, there was a predominance of more advanced stages of endometriosis. Further studies on different regions and ethnic groups seem necessary to assess the effects of the changes in these polymorphisms with the etiology of endometriosis.

## CONCLUSION

In this study, our results show that the studied polymorphisms are not implicated in the development of endometriosis.
